# Moisture-driven shifts in the fermentation characteristics and microbial community of alfalfa silage treated with different additives

**DOI:** 10.3389/fvets.2026.1748640

**Published:** 2026-02-27

**Authors:** Run Gao, Bo Wu, Zhiqiang Sun, Zhu Yu, Chunlin Jia, Daniel Basigalup, Guoliang Wang

**Affiliations:** 1 Institute of Leisure Agriculture, Shandong Academy of Agricultural Sciences, Jinan, China; 2 College of Grassland Science and Technology, China Agricultural University, Beijing, China; 3 National Institute of Agricultural Technology (INTA), Centro Regional Córdoba, Argentina

**Keywords:** additives, alfalfa silage, bacterial community, fermentation characteristics, moisture contents

## Abstract

Understanding the changes in the bacterial ecosystem during anaerobic fermentation of alfalfa (*Medicago sativa* L.) could provide clearer insight into how moisture content and additives affect the fermentation characteristics and chemical composition of alfalfa silage. Alfalfa was harvested at the budding stage, with moisture contents of 78, 68, and 58%. The treatments included a control (CK), commercial *Lactiplantibacillus plantarum* (CL), screened *L. plantarum* (LP, 1 × 10^6^ cfu/g FW), and propionic acid (P, 6 mL/kg FW). The results showed that the addition of CL, LP, and P significantly reduced pH and NH_3_-N content and decreased the relative abundance of *Enterococcus* at moisture contents of 78 and 68%. These treatments also increased LA and dry matter (DM) content and decreased the relative abundance of *Lactobacillus*. The addition of CL and LP significantly reduced pH, NH_3_-N content, and the relative abundance of *Enterococcus*, while increasing the relative abundance of *Lactobacillus* at a moisture content of 58%. Functional prediction analysis suggested that additives improved metabolism functions in alfalfa silage. Carbohydrate metabolism, specifically starch and sucrose metabolism, accounted for the highest proportion in the LP treatment group. *Lactobacillus* and *Sphingomonas* were negatively correlated with pH and positively correlated with LA and Flieg’s score, whereas *Enterococcus*, *Weissella*, *Leuconostoc*, and *Enterobacter* were positively correlated with pH. In conclusion, the absence of wilting was not conducive to the anaerobic fermentation of alfalfa. Appropriately reducing the moisture content was beneficial for enhancing the effectiveness of additives in promoting alfalfa fermentation.

## Introduction

“Increasing forage and saving food” is an important strategy to ensure food security, accelerate the high-quality development of animal husbandry, reduce the consumption of concentrated feeds, and promote sustainable agricultural development (1). Alfalfa (*Medicago sativa* L.) is a widely used perennial legume forage worldwide, known as the “queen of forages.” It contains high levels of crude protein (CP), calcium, and phosphorus; is rich in all essential amino acids required for animal growth; and is characterized by its abundance of vitamins and mineral elements (2). In addition, alfalfa can be harvested multiple times each year and is characterized by high productivity. It plays a crucial role in dairy farming and other animal husbandry systems (3, 4) and can be cultivated under various environmental conditions due to its extensive adaptability (5).

More than 70% of the Yellow River Delta region consists of saline-alkali soil (6). In recent years, the planting area of alfalfa in this region has increased, making it an important region for the development of alfalfa (7). Nevertheless, the harvest time of alfalfa often coincides with the rainy season (8), which makes wilting difficult, seriously reduces the quality of alfalfa (9), and poses a potential threat to the growth of livestock. Silage is an effective method for storing forage through anaerobic fermentation, which not only prolongs the storage period but also preserves its nutritional components to a certain extent and improves its digestibility (10). However, alfalfa presents significant ensiling challenges due to its high moisture content, high buffering capacity, and low water-soluble carbohydrate content (11). These characteristics often result in restricted acidification and unstable fermentation, leading to protein degradation and dry matter (DM) losses. The initial moisture content is a critical determinant of ensiling success, profoundly influencing microbial activity, fermentation pathways, and the final silage quality (12). Excessively high moisture can promote clostridial fermentation and cause effluent losses, while excessively low moisture may hinder compaction and sufficient fermentation. Therefore, identifying the optimal moisture range for specific forages, such as alfalfa, is essential.

To improve fermentation efficiency and silage quality, various microbial inoculants and chemical additives are used to promote anaerobic fermentation of alfalfa (13, 14). Lactic acid bacteria inoculants are commonly used to dominate fermentation, rapidly producing lactic acid and accelerating the pH decline (15). Studies have shown that adding *Lactiplantibacillus plantarum* (LP) and cellulase can improve the quality of alfalfa mixed with *Leymus chinensis* silage (16). Although commercial *L. plantarum* strains are effective, recent research has explored the potential of screened *L. plantarum* strains that may be better adapted to specific forage conditions. In contrast, chemical additives, such as propionic acid (P), primarily function as potent inhibitors of yeasts and molds, enhancing aerobic stability upon silage exposure to air (17). However, the interactive effects of critical moisture levels and different types of additives—comparing screened versus commercial bacteria and biological versus chemical modes of action—on the overall silage profile have not been fully elucidated.

Modern high-throughput sequencing technologies have revolutionized our understanding of silage microbiology, moving beyond culture-dependent methods. Investigating the composition of the bacterial community and its metabolic functional potential is crucial for explaining observed fermentation patterns. Furthermore, elucidating the correlations between dominant bacterial taxa and key fermentation and nutritional indicators can reveal the microbial drivers of silage quality. The composition of the microbial community in silage can provide a comprehensive indication of silage quality (18). Superior silage is primarily led by lactic acid bacteria (19), whereas low-quality silage with complex microbial compositions is predominantly dominated by undesirable bacteria, such as *Enterobacter*, *Clostridium*, *Listeria,* and *Salmonella* (20). In such silage, beneficial bacteria such as lactic acid bacteria often represent only a small proportion of the microbial community (21).

Therefore, this study was designed to explore the effects of three distinct moisture levels (78, 68, and 58%) and two LP inoculants and P on alfalfa silage. We comprehensively evaluated the fermentation characteristics, chemical composition, and microbial community dynamics. The objectives of this study were to: (1) determine the optimal moisture content for alfalfa ensiling, (2) assess and compare the efficacy of different additives under varying moisture conditions, (3) analyze shifts in bacterial community structure and predict potential microbial functions, and (4) identify key bacterial taxa significantly associated with crucial fermentation and nutritional parameters. This integrated approach aims to provide insights for developing targeted strategies to enhance the preservation and quality of alfalfa silage.

## Materials and methods

### Alfalfa ensiling

Alfalfa (*Medicago sativa L.*) was harvested from the Yellow River Delta Modern Agriculture Experimental Demonstration Base of the Shandong Academy of Agricultural Sciences, located in the Nonggao district of Dongying City (37°18′N, 118°37′E) at an altitude of 2 m. The region has a warm temperate monsoon climate. The alfalfa variety used was WL440, and the cutting period was the end of the fourth budding stage, with a stubble height of 5 cm. The moisture content of alfalfa was adjusted to three levels: (1) approximately 78%, without wilting; (2) approximately 68%, wilted for almost 2 h; and (3) approximately 58%, wilted for almost 4 h. Real-time moisture measurements were performed using a microwave oven. After wilting, the alfalfa was chopped into 1–2 cm pieces using a chaff cutter (9Z-0.4, Jinniu Machinery Factory, Rongyang, Henan Province). A total of three replicates of alfalfa at each moisture content were reserved for measuring dry matter (DM) and CP contents. In total, four additive treatment groups were established for each moisture content of alfalfa: (1) Blank control (CK, with an equal amount of distilled water), (2) commercial *L. plantarum* (1 × 10^6^ cfu/g FW, CL, Yiqing No. 2, purchased from Beijing Zhongqing Yicao Technology Co., Ltd.), (3) *L. plantarum* (1 × 10^6^ cfu/g FW, LP, screened from sheep skin soaking solution by the Grass Product Processing Laboratory of China Agricultural University), and (4) propionic acid (P, applied at 6 mL/kg FW, diluted to a concentration of 45%). The alfalfa was thoroughly mixed with the additives, and approximately 200 g of the mixture was placed into polyethylene vacuum bags. The bags were sealed using a vacuum packaging machine. In total, 3 moisture levels * 4 additive treatments * 3 replicates resulted in 36 silage bags. All bags were stored at 25 °C (room temperature) and opened after 45 days of ensiling.

### Fermentation characteristic analysis

On the day of opening, a 10 g sample was collected and mixed with 90 mL of distilled water, then homogenized using a liquidizer for 1 min. The mixture was subsequently filtered through four layers of sterile gauze followed by qualitative filter paper. The pH was measured using a LEI-CI PHS-3C pH meter (Shanghai Yidian Scientific Instrument Co., Ltd., Shanghai, China). As previously described (22), the contents of lactic acid (LA), acetic acid (AA), propionic acid (PA), and butyric acid (BA) were determined usingHPLC. The ammonia nitrogen (AN) content was measured following the method described by Broderick and Kang (23). Flieg’s score was used to evaluate the fermentation quality of alfalfa silage, calculated as follows (24): Flieg’s score = 220 + (2 × %DM − 15) − 40 × pH.

### Dry matter and crude protein content analysis

The DM content was measured as previously reported (9). Samples were dried at 65 °C for 48 h in a heating and drying oven (LC-202-00, Shanghai Lichen Instrument Technology Co., Ltd., Shanghai, China). The CP content was measured using the dried samples, which were ground with a grinder (BJ-800A, Deqing Baijie Electric Appliance Co., Ltd., Huzhou, Zhejiang, China) and passed through a 1 mm sieve. The CP content was calculated as total nitrogen (TN) multiplied by 6.25. TN was determined using an automatic Kjeldahl nitrogen apparatus (Kjeltec 2300 AutoAnalyzer, FOSS Analytical AB, Hoganas, Sweden) according to the AOAC (25) standard.

### Microbial DNA extraction, PCR amplification, and sequencing analysis

Microbial genomic DNA was extracted from ensiled alfalfa using the E.Z.N.A.® Soil DNA Kit (Omega Bio-tek, Norcross, GA, USA) according to the manufacturer’s instructions. Then, 1.0% agarose gel electrophoresis and a NanoDrop® ND-2000 spectrophotometer (Thermo Scientific Inc., Waltham, MA, USA) were used to assess the quality and concentration of the DNA, and the DNA was stored at −80 °C until further analysis. The primer pair 338F (5′-ACTCCTACGGGAGGCAGCAG-3′) and 806R (5′-GGACTACHVGGGTWTCTAAT-3′) was used to amplify the bacterial 16S rRNA gene hypervariable region V3-V4 on an ABI GeneAmp® 9700 PCR thermocycler (ABI, Foster City, CA, USA). PCR was performed following the previously described method (26). The PCR product was extracted using 2% agarose gel, purified using the AxyPrep DNA Gel Extraction Kit (Axygen Biosciences, Union City, CA, USA) according to the manufacturer’s instructions, and quantified using a Quantus™ Fluorometer (Promega, USA). Purified amplicons were pooled in equimolar amounts and sequenced using paired-end reads on an Illumina MiSeq PE300 platform (Illumina, San Diego, USA) according to the standard protocols by Majorbio Bio-Pharm Technology Co., Ltd. (Shanghai, China). Raw FASTQ files were de-multiplexed using an in-house Perl script, quality-filtered with fastp version 0.19.6, and merged using FLASH version 1.2.7. Then, the optimized sequences were clustered into operational taxonomic units (OTUs) at 97% sequence similarity using UPARSE 7.1.

### Statistical analyses

The experiment was conducted as a completely randomized design with a 3 × 4 (three moisture levels and four additives) factorial arrangement. Analyses of fermentation characteristics and chemical composition were performed using the SAS software (version 9.4, SAS Institute Inc., Cary, NC, USA). A mixed model procedure (GLM) was used as described by Gao et al. (21) as follows:


$$Y_{\mathrm{ijk}}=\mu +M_{i}+A_{j}+{\left(M\times A\right)}_{\mathrm{ij}}+e_{\mathrm{ijk}}$$

Where *Y_ijk_* is the dependent variable representing the response for the moisture content *i* observed under additive *j*, *μ* is the mean, *M_i_* is the moisture content effect, *A_j_* is the additive effect, (*M* × *A*)*_ij_* is the interaction effect of moisture content and additive, and *e_ijk_* is the residual error.

ANOVA was used to evaluate the effects of additives at each moisture level or the effects of moisture levels for each additive. The model used was as follows:


$$Y=\mu +M_{i}+e_{\mathrm{ij}}\;\text{or}\;Y=\mu +A_{i}+e_{\mathrm{ij}}$$

Where *Y* is the response variable, *μ* is the overall mean, *M_i_* is the effect of the moisture content, *A_i_* is the effect of the additive, and *ε_ij_* is the residual error. *Post hoc* mean comparisons were performed using Tukey’s multiple range test. Statistical significance was set at a *p*-value of ≤0.05. Microbial data analysis was conducted on the Majorbio Bio-Pharm Technology Co., Ltd. (Shanghai, China) cloud platform.^1^

## Results

### Dry matter and crude protein content of alfalfa before ensiling

The moisture content of alfalfa without wilting was 78.31%; after 2 h of wilting, it was 67.32%; and after 4 h of wilting, it was 59.73%. The crude protein (CP) content before ensiling was 23.32%.

### Fermentation characteristics and chemical composition of alfalfa silage

Moisture had a significant impact on pH, AA, AN, Flieg’s score, DM, and CP (*p* < 0.05). The additive significantly affected pH, LA, PA, BA, AN, Flieg’s score, DM, and CP (*p* < 0.05). Moreover, the interaction between moisture and additive had a significant effect on pH, LA, PA, BA, AN, Flieg’s score, DM, and CP (*p* < 0.05) in alfalfa silage (Table 1).

**Table 1:** Effects of moisture levels, additives, and their interaction on the fermentation characteristics and chemical composition of alfalfa silage.

Index	Interaction *p*-value		
	*M*	*A*	*M* × *A*
pH	<0.001	<0.001	<0.001
LA	0.177	<0.001	<0.001
AA	0.029	0.988	0.141
PA	0.108	<0.001	0.006
BA	0.090	0.046	0.017
LA:AA	0.247	0.027	0.145
AN	<0.001	<0.001	0.014
Flieg’s score	<0.001	<0.001	<0.001
DM	<0.001	<0.001	0.018
CP	<0.001	0.001	0.004

The effects of the moisture contents and additives on the fermentation characteristics of alfalfa silage are shown in Table 2. The pH of the P group at the 78% moisture content was significantly lower than that of the other treatment groups (*p* < 0.05). The CL, LP, and P treatments at the 68% moisture content had significantly lower pH values than the CK group (*p* < 0.05). At the 58% moisture content, the pH of the P group was significantly higher than that of the CK, CL, and LP groups (*p* < 0.05). At the 78% moisture content, the LA contents of the CL and P groups were significantly higher than those of the CK and LP groups (*p* < 0.05). At the 68% moisture content, the LA contents of the LP and P groups were significantly higher than those of the CK and CL groups (*p* < 0.05). At the 58% moisture content, the LA contents of the CL and LP groups were significantly higher than those of the CK and P groups (*p* < 0.05). The LA content of the CL group at 78 and 58% moisture contents was significantly higher than that at the 68% moisture content (*p* < 0.05). In the P group, the LA content at 78 and 68% moisture contents was significantly higher than that at the 58% moisture content (*p* < 0.05). The PA content of the P group was significantly higher than that of the CK and LP groups at the 78% moisture content (*p* < 0.05) and higher than that of the other groups at the 68% moisture content (*p* < 0.05). The AN content of alfalfa silage showed a decreasing trend with decreasing moisture content. The AN content of the CK group was significantly higher than that of the other groups at 78 and 58% moisture contents (*p* < 0.05). The Flieg’s score of the P group was significantly higher than that of the CL, LP, and CK groups at the 78% moisture content (*p* < 0.05). The CK group had a significantly lower Flieg’s score than the other groups at the 68% moisture content (*p* < 0.05). The Flieg’s score of the CL and LP groups was significantly higher than that of the CK and P groups at the 58% moisture content (*p* < 0.05).

**Table 2:** Effects of moisture content and additives on the fermentation characteristics of alfalfa silage.

Items	Moisture content	Additives				*p*-value
		CK	CL	LP	P	
pH	78%	5.54 ± 0.02Aa	4.81 ± 0.01Ab	4.87 ± 0.02Ab	4.53 ± 0.01Bc	<0.001
	68%	5.49 ± 0.02Aa	4.37 ± 0.01Bb	4.37 ± 0.01Bb	4.37 ± 0.01Cb	<0.001
	58%	5.27 ± 0.02Bb	4.29 ± 0.00Cc	4.34 ± 0.01Bc	5.38 ± 0.04Aa	<0.001
	*p*-value	0.006	<0.001	<0.001	<0.001	
LA	78%	1.83 ± 0.13b	3.88 ± 0.33Aa	2.87 ± 0.84ab	4.50 ± 0.21a	0.027
	68%	1.91 ± 0.15b	2.34 ± 0.08Bb	3.71 ± 0.34a	4.48 ± 0.37a	0.027
	58%	1.64 ± 0.11b	3.94 ± 0.08Aa	3.55 ± 0.37a	2.17 ± 0.21b	0.001
	*p*-value	>0.05	0.009	>0.05	>0.05	
AA	78%	2.62 ± 0.47	2.40 ± 0.22A	1.83 ± 0.56	1.95 ± 0.32	>0.05
	68%	1.48 ± 0.12	1.23 ± 0.03B	1.77 ± 0.14	1.92 ± 0.43	>0.05
	58%	1.31 ± 0.36	2.02 ± 0.08A	1.98 ± 0.24	1.73 ± 0.21	>0.05
	*p*-value	>0.05	0.012	>0.05	>0.05	
PA	78%	1.22 ± 0.23b	1.95 ± 0.13Aab	1.37 ± 0.39b	2.70 ± 0.23Aa	0.034
	68%	1.63 ± 0.18b	1.04 ± 0.06Cc	1.32 ± 0.10bc	2.68 ± 0.06Aa	<0.001
	58%	1.34 ± 0.17	1.42 ± 0.01B	1.49 ± 0.15	1.83 ± 0.17B	>0.05
	*p*-value	>0.05	0.006	>0.05	>0.05	
BA	78%	0.02 ± 0.01a	0.00 + 0.00b	0.01 ± 0.00Aa	0.00 + 0.00b	0.044
	68%	0.01 ± 0.00	0.01 ± 0.00	0.00 + 0.00B	0.01 ± 0.00	>0.05
	58%	0.01 ± 0.00	0.00 + 0.00	0.00 + 0.00B	0.00 + 0.00	>0.05
	*p*-value	>0.05	>0.05	0.010	>0.05	
AN	78%	10.51 ± 0.84Aa	6.49 ± 0.46Ab	6.07 ± 0.57Ab	4.64 ± 0.12Ac	<0.001
	68%	4.42 ± 1.22B	2.67 ± 0.58B	1.70 ± 0.06B	2.28 ± 0.25B	>0.05
	58%	4.02 ± 0.25Ba	1.21 ± 0.04Bb	1.41 ± 0.19Bb	1.81 ± 0.05Bb	<0.001
	*p*-value	0.005	0.005	0.001	<0.001	
Flieg’s score	78%	23.66 ± 1.13Cc	54.70 ± 0.97Cb	52.53 ± 1.25Cb	67.03 ± 0.60Ca	<0.001
	68%	48.92 ± 0.62Bb	96.20 ± 0.58Ba	96.28 ± 0.26Ba	95.43 ± 0.29Aa	<0.001
	58%	73.24 ± 0.86Ab	113.05 ± 0.25Aa	111.92 ± 0.57Aa	71.21 ± 2.09Bb	<0.001
	*p*-value	<0.001	<0.001	<0.001	<0.001	

Different capital letters indicates that the same additive with different moisture contents showed a significant difference (*p* < 0.05). Different lowercase letters indicates that different additives showed a significant difference among each other (*p* < 0.05).

The effects of different moisture contents and additives on the DM and CP content of alfalfa silage are shown in Table 3. The DM of the P group was significantly higher than that of the CK and CL groups at the 78% moisture content (*p* < 0.05). The DM of the CL, LP, and P groups was significantly higher than that of the CK group at the 68% moisture content (*p* < 0.05). The DM of the LP and P groups was significantly higher than that of the CK group at the 58% moisture content (*p* < 0.05). The CP content of the CL, LP, and P groups was significantly higher than that of the CK group at the 68% moisture content (*p* < 0.05).

**Table 3:** Effects of moisture content and additives on the DM and CP contents of alfalfa silage.

Items	Additives	Moisture content			*p*-value
		78%	68%	58%	
DM (%)	CK	20.06 ± 0.06Cc	31.83 ± 0.06Bb	39.52 ± 0.04Ac	<0.001
	CL	21.05 ± 0.07Cb	33.07 ± 0.04Ba	39.89 ± 0.04Abc	<0.001
	LP	21.10 ± 0.06Cab	32.97 ± 0.01Ba	40.33 ± 0.03Aab	<0.001
	P	21.62 ± 0.04Ca	32.68 ± 0.06Ba	40.71 ± 0.06Aa	<0.001
	*p*-value	0.002	0.001	0.013	
CP (%DM)	CK	20.75 ± 0.39B	20.59 ± 0.37Bb	23.30 ± 0.57A	0.002
	CL	21.61 ± 0.45B	22.36 ± 0.45ABa	23.12 ± 0.39A	0.048
	LP	22.09 ± 0.43	22.81 ± 0.38a	23.40 ± 0.66	>0.05
	P	22.17 ± 0.25B	22.76 ± 0.2Aa	22.36 ± 0.21B	0.004
	*p*-value	>0.05	<0.001	>0.05	

Different capital letters indicates that the same additive with different moisture contents showed a significant difference (*p* < 0.05). Different lowercase letters indicates that different additives showed a significant difference among each other (*p* < 0.05).

### Bacterial diversity and community composition of alfalfa silage

**Figure 1:**
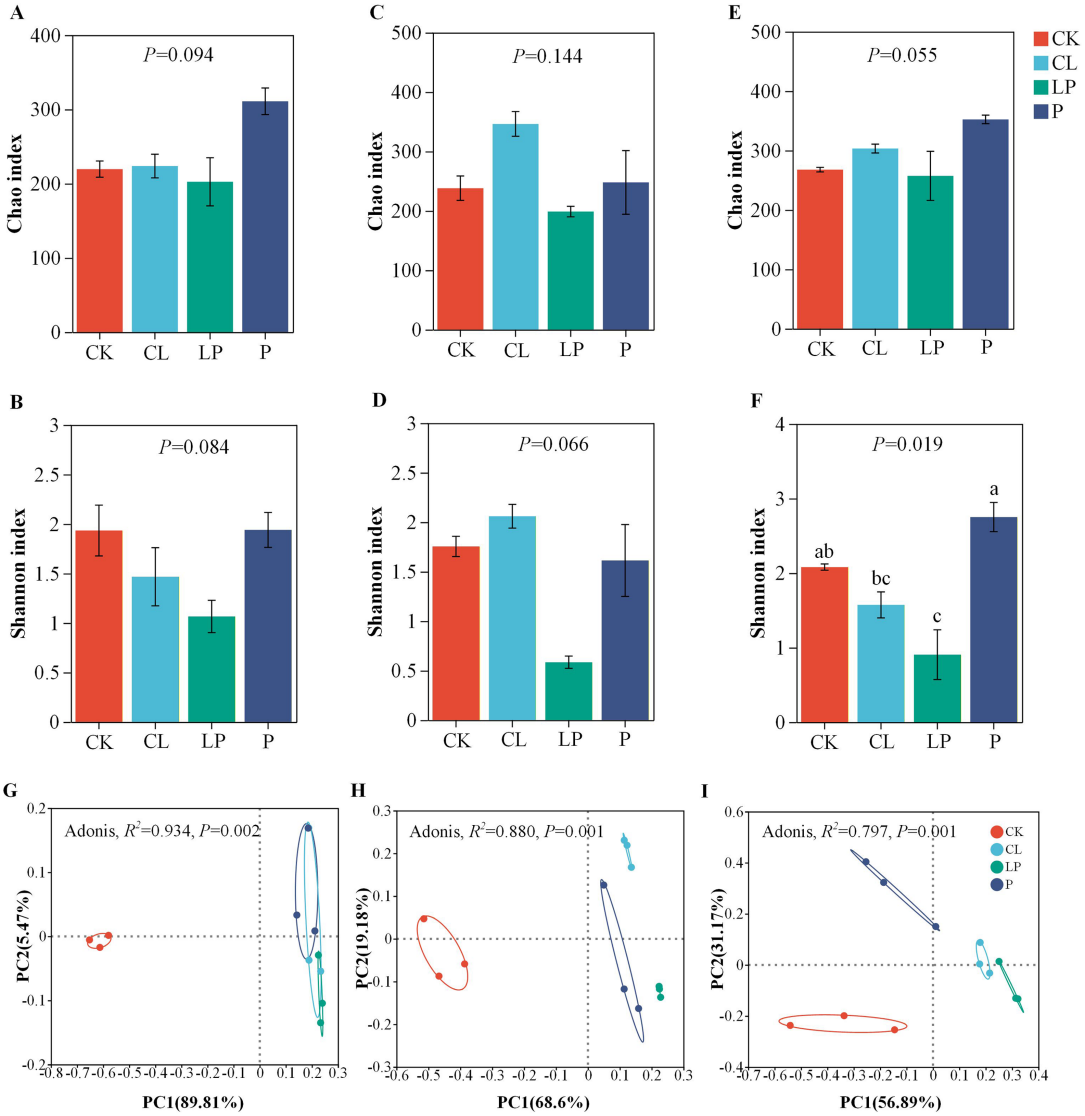
Alpha and beta diversity of alfalfa silage among the four additive groups at different moisture contents. **(A,B)** Alpha diversity (Chao and Shannon indices) of bacterial communities among the four additive groups at the 78% moisture content. **(C,D)** Alpha diversity (Chao and Shannon indices) of bacterial communities among the four additive groups at the 68% moisture content. **(E,F)** Alpha diversity (Chao and Shannon indices) of bacterial communities among the four additive groups at the 58% moisture content. Beta diversity based on principal coordinate analysis (PCoA) using Bray–Curtis dissimilarity matrices among the four additive groups at 78% **(G)**, 68% **(H)**, and 58% **(I)** moisture contents. CK: Control group with an equal amount of distilled water; CL: Group inoculated with commercial *Lactobacillus plantarum*; LP: Group inoculated with *L. plantarum*; and P: Group treated with propionic acid. PC1, principal coordinate 1; PC2, principal coordinate 2.

The alpha and beta diversity of alfalfa silage among the four additive groups showed no significant difference at ecah moisture contents (Figures 1A–E), except for Shannon index at the 58% moisture content (Figure 1F). Alpha diversity analysis revealed that the LP group of alfalfa silage had a significantly lower Shannon index than the CK and P groups at the 58% moisture content (*p* < 0.05). A marked divergence in the Shannon index among the four additive treatments at the 58% moisture content indicated that the LP treatment reduced the diversity of certain microbial communities. Principal coordinates analysis (PCoA) demonstrated clear separation among the additive groups at each moisture content (Figures 1G–I), indicating that different additives caused marked differences in bacterial community composition at each moisture content.

**Figure 2:**
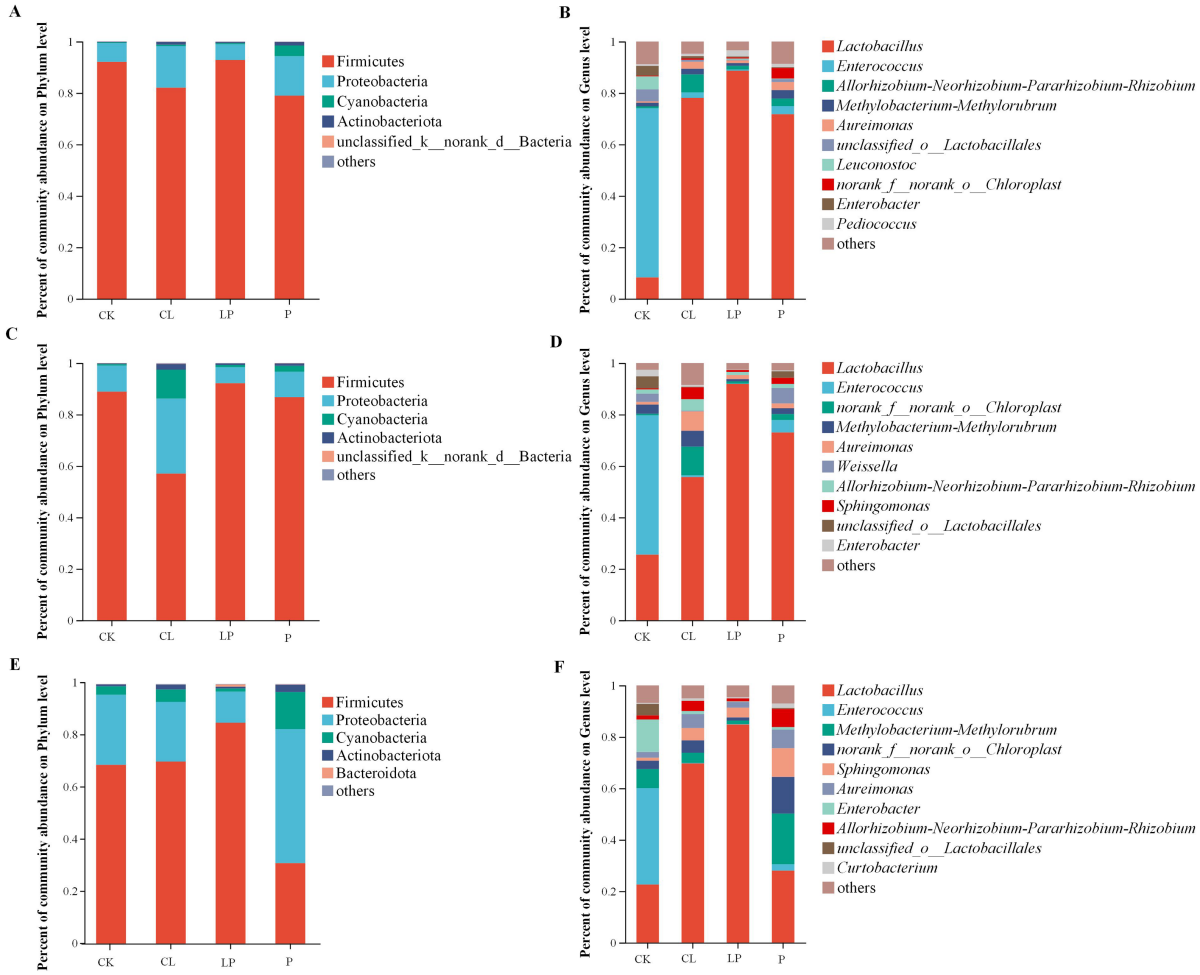
Bar plots showing the bacterial community composition of alfalfa silage at 78, 68, and 58% moisture contents. **(A,B)** Relative abundance of the bacterial community at the phylum and genus levels among the four additive groups at the 78% moisture content. **(C,D)** Relative abundance of the bacterial community at the phylum and genus levels among the four additive groups at the 68% moisture content. **(E,F)** Relative abundance of the bacterial community at the phylum and genus levels among the four additive groups at the 58% moisture content. CK: Group with an equal amount of distilled water; CL: Group inoculated with commercial *Lactobacillus plantarum*; LP: Group inoculated with *L. plantarum*; and P: Group treated with propionic acid.

**Figure 3:**
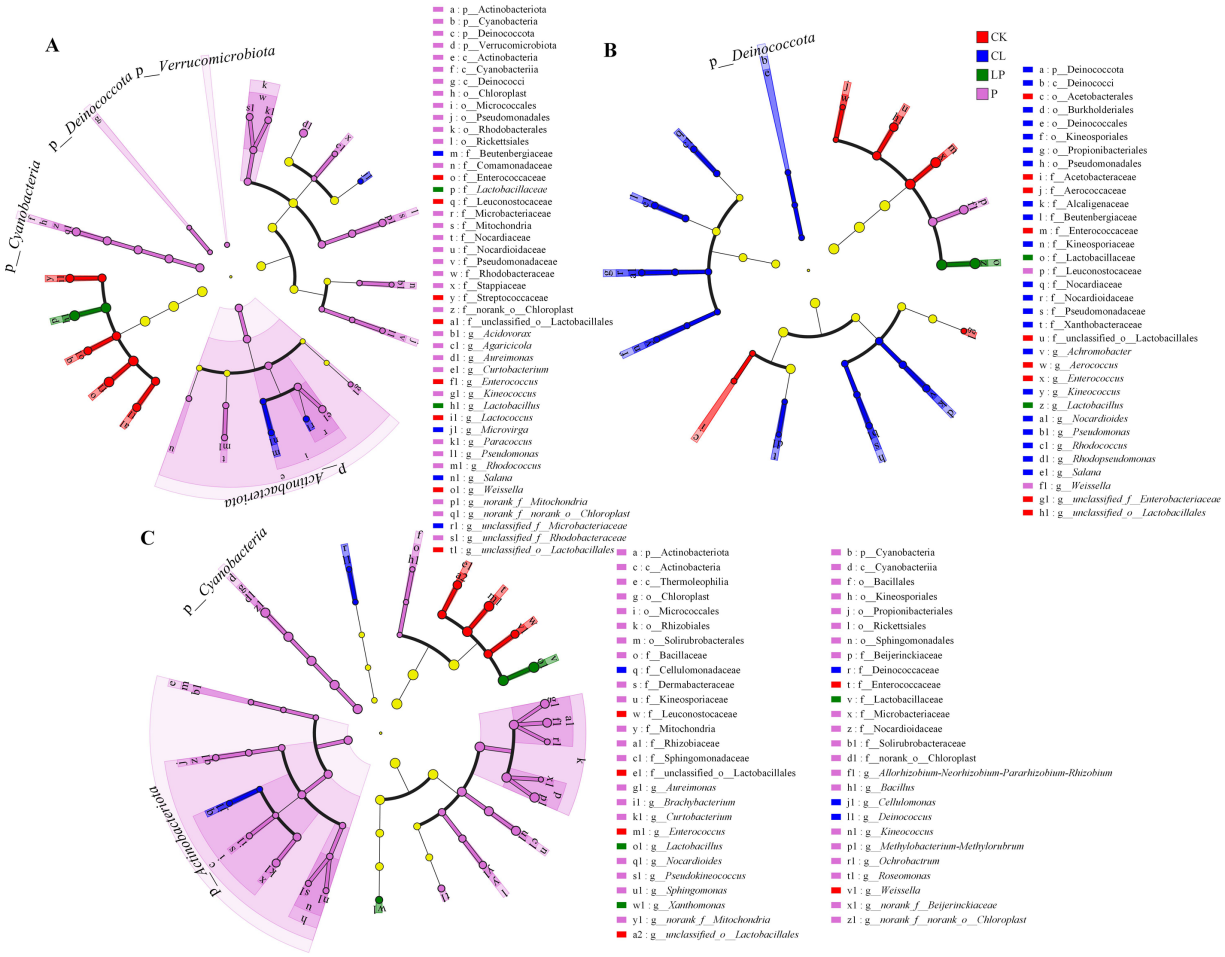
Differential bacterial taxa among the four additive groups at different taxonomic levels. Significantly different bacterial taxa among the four additive groups at 78% **(A)**, 68% **(B)**, and 58% **(C)** moisture contents. Different colored nodes represent bacteria that were significantly enriched in their corresponding additive groups and had a significant impact on the microbial community. The yellow nodes indicate bacteria that showed no significant differences among the different additive groups or had no significant impact on the microbial community. CK: Group with an equal amount of distilled water; CL: Group inoculated with commercial *Lactobacillus plantarum*; LP: Group inoculated with *L. plantarum*; and P: Group treated with propionic acid.

At the phylum level, the predominant phyla across the 36 samples were Firmicutes and Proteobacteria (Figures 2A,C,E; Supplementary Table S1). Cyanobacteria, Actinobacteriota, and Deinococcota showed significant differences among the four groups at the 78% moisture content. Deinococcota showed significant differences among the four groups at the 68% moisture content. Actinobacteriota, Cyanobacteria, and Verrucomicrobiota showed significant differences among the four groups at the 58% moisture content (Figures 3A–C; Supplementary Table S2).

At the genus level, the dominant genera across the 36 samples were *Lactobacillus* and *Enterococcus* (Figures 2B,D,F; Supplementary Table S2). Among the four treatment groups, 19 genera showed significant differences at the 78% moisture content, 13 at the 68% moisture content, and 37 at the 58% moisture content (Figure 3). The abundance of *Lactobacillus* was higher in the additive groups compared to the CK group, whereas the abundance of *Enterococcus* was lower in the additive groups compared to the CK group (Figure 2; Supplementary Table S2). The abundance of *Lactobacillus* was lower in the P group at the 58% moisture content, while *Methylobacterium-Methylorubrum*, *norank_f__norank_o__Chloroplast*, *Sphingomonas*, *Aureimonas,* and *Allorhizobium-Neorhizobium-Pararhizobium-Rhizobium* together accounted for more than 59% of the bacterial community (Supplementary Table S2). *Enterococcus* was the dominant genus in the CK group at all moisture contents.

### Microbiota functions of alfalfa silage

At KEGG pathway level 1, six categories—metabolism, environmental information processing, genetic information processing, human diseases, cellular processes, and organismal systems—were identified across the 36 samples at each moisture content of alfalfa silage (Supplementary Figure S1). The metabolism pathway was the most active (Table 4). There was a significant difference between the CK group and the CL group at the 78% moisture content. A significant difference was observed between the CK group and the LP group at the 68% moisture content. At the 58% moisture content, the CK group showed a significant difference compared to the LP group, and the P group was significantly different from the CL and LP groups.

**Table 4:** Predicted metabolic functions of alfalfa silage treated with different additives at varying moisture contents.

Moisture content	CK	CL	LP	P	*p-*value
78%	75.17 ± 0.48	76.38 ± 0.85	76.84 ± 0.15	76.29 ± 0.35	0.092
68%	75.89 ± 0.51	76.22 ± 0.33	77.10 ± 0.06	76.59 ± 0.40	0.043
58%	75.77 ± 0.45	76.51 ± 0.37	76.93 ± 0.33	75.47 ± 0.36	0.040

At KEGG pathway level 2, carbohydrate metabolism and amino acid metabolism were the most active pathways (Supplementary Figure S2). The LP group showed higher carbohydrate metabolism activity in alfalfa silage at all moisture contents (Table 5), which was significantly higher than that of the CK group at the 78% moisture content, the CL group at the 68% moisture content, and the P group at the 58% moisture content (Supplementary Figure S3). The additives promoted amino acid metabolism in alfalfa silage (Table 5). The amino acid metabolism activity of the LP group was significantly higher than that of the CK group at the 78% moisture content and the CL group at the 68% moisture content. The CL and LP groups showed significantly higher amino acid metabolism activity than the P group at the 58% moisture content (Supplementary Figure S3).

**Table 5:** Functional predictions of carbohydrate and amino acid metabolism in alfalfa silage treated with different additives at different moisture contents.

Moisture content	Names	CK	CL	LP	P	*p-*value
78%	Carbohydrate metabolism	11.91 ± 0.4	12.1 ± 0.83	12.7 ± 0.25	11.87 ± 0.53	0.154
	Amino acid metabolism	5.65 ± 0.31	6.55 ± 0.06	6.38 ± 0.11	6.36 ± 0.02	0.025
68%	Carbohydrate metabolism	12.1 ± 0.58	11.02 ± 0.13	12.78 ± 0.08	12.27 ± 0.75	0.038
	Amino acid metabolism	6.21 ± 0.16	6.75 ± 0.15	6.55 ± 0.02	6.36 ± 0.12	0.022
58%	Carbohydrate metabolism	11.08 ± 0.84	11.74 ± 0.35	12.44 ± 0.55	9.66 ± 0.9	0.049
	Amino acid metabolism	6.45 ± 0.08	6.69 ± 0.12	6.61 ± 0.12	6.86 ± 0.04	0.027

At KEGG pathway level 3, the top pathway for carbohydrate metabolism was starch and sucrose metabolism, while the top pathway for amino acid metabolism was cysteine and methionine metabolism (Supplementary Figure S5). No significant difference was observed in starch and sucrose metabolism at the 78% moisture content. A significant difference between the CL and LP groups was observed at the 68% moisture content. Starch and sucrose metabolism in the P group was significantly higher than that in the CL and LP groups at the 58% moisture content (Table 6). Cysteine and methionine metabolism in the LP group was significantly higher than that in the CK group at the 78% moisture content. The LP group showed higher cysteine and methionine metabolism than the CK and CL groups at the 68% moisture content. Furthermore, cysteine and methionine metabolism in the LP group was significantly higher than that in the CK and P groups at the 58% moisture content, and it was also significantly higher in the CL group compared to the P group (Table 6).

**Table 6:** Functional predictions of carbohydrate and amino acid metabolism in alfalfa silage treated with different additives at different moisture contents under KEGG pathway level 3.

Moisture content	Names	CK	CL	LP	P	p*-*value
78%	Starch and sucrose metabolism	1.78 ± 0.09	1.92 ± 0.38	2.18 ± 0.09	1.83 ± 0.21	0.147
	Cysteine and methionine metabolism	0.89 ± 0.04	1.06 ± 0.13	1.12 ± 0.02	1.01 ± 0.06	0.121
68%	Starch and sucrose metabolism	1.9 ± 0.24	1.56 ± 0.04	2.24 ± 0.03	2.01 ± 0.28	0.022
	Cysteine and methionine metabolism	1.02 ± 0.07	1.01 ± 0.02	1.18 ± 0.01	1.08 ± 0.07	0.063
58%	Starch and sucrose metabolism	1.54 ± 0.35	1.83 ± 0.16	2.12 ± 0.21	1.06 ± 0.32	0.034
	Cysteine and methionine metabolism	0.97 ± 0.09	1.07 ± 0.05	1.15 ± 0.05	0.88 ± 0.07	0.029

### Analysis of the correlation between bacterial diversity, fermentation characteristics, and chemical composition

**Figure 4:**
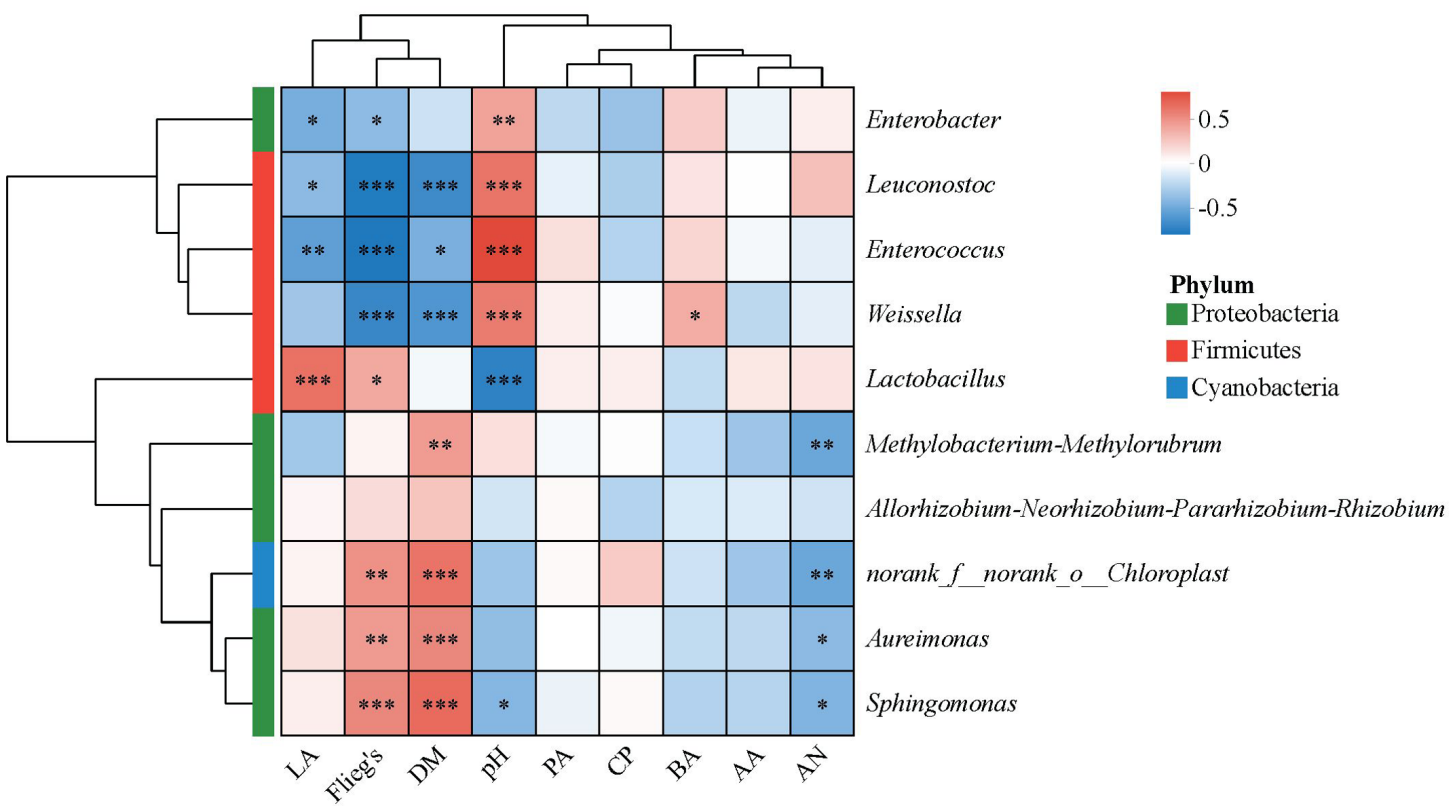
Spearman correlation heatmap of bacterial diversity, fermentation characteristics, and chemical composition at the genus level. Positive correlations are shown in red, and negative correlations are shown in blue. ^*^means 0.01 < *p* ≤ 0.05, ^**^means 0.001 < *p* ≤ 0.01, ^***^means *p* ≤ 0.001.

The Spearman correlation between bacterial diversity, fermentation characteristics, and chemical composition is shown in Figure 4. It was found that pH was negatively correlated with *Lactobacillus* and *Sphingomonas* and positively correlated with *Enterococcus*, *Weissella*, *Leuconostoc*, and *Enterobacter*. The LA content was negatively correlated with *Enterococcus*, *Leuconostoc*, and *Enterobacter* and positively correlated with *Lactobacillus*. The AN content was negatively correlated with *Methylobacterium-Methylorubrum*, *Sphingomonas*, and *Aureimonas*. The BA content was positively correlated with *Weissella*. Flieg’s score was negatively correlated with *Enterococcus*, *Weissella*, *Leuconostoc*, *and Enterobacter* and positively correlated with *Sphingomonas*, *Aureimonas*, and *Lactobacillus*. The DM content was negatively correlated with *Weissella*, *Leuconostoc*, *and Enterococcus* and positively correlated with *Sphingomonas*, *Aureimonas*, and *Methylobacterium-Methylorubrum*.

## Discussion

The moisture content of forage before ensiling is a key factor in determining silage quality (27). In our study, alfalfa was ensiled at three different moisture contents: 78, 68, and 58%. The pH is an important indicator for evaluating the fermentation quality of silage (28). In our study, the pH of alfalfa silage decreased as the moisture content decreased, indicating that wilting promotes lactic acid bacteria fermentation and inhibits the growth of harmful microorganisms in alfalfa silage. It has been reported that wilting helps additives to reduce the pH of alfalfa silage (29). A previous study showed that alfalfa silage with a moisture content of 60% had a lower pH than non-wilted alfalfa silage, which is consistent with the results of our study (14). All additives reduced the pH of alfalfa silage at each moisture content, except for the P group at the 58% moisture content. This may be due to a decrease in beneficial microorganisms and an increase in harmful microorganisms on the surface of the alfalfa after prolonged wilting. This indicates that the P treatment had a noticeable effect on alfalfa silage when the moisture content was higher. Lactic acid bacteria additives significantly reduced the pH of alfalfa silage at all moisture contents. A possible reason is that high-temperature wilting not only reduced the moisture content of alfalfa but also inactivated beneficial lactic acid bacteria on the surface of alfalfa, leading to silage spoilage. It was found that adding P could significantly reduce the pH of oat silage with a moisture content of 74.55% (after 24 h of wilting) (17), which is consistent with the result of our study. In addition, the LA content showed a trend opposite to that of pH, further indicating that wilting enhanced the effect of additives on the fermentation quality of alfalfa. A study reported that alfalfa silage without additives at the 72% moisture content had a higher LA content than at the 53% moisture content (29); however, this difference was not significant in our results. Furthermore, CL-treated alfalfa silage at the 68% moisture content showed lower AA content compared to 78 and 58% moisture contents, indicating that wilting may inhibit the fermentation of AA-producing bacteria (14). It has also been reported that organic acids are less effective than lactic acid bacteria treatments (14). In our study, the addition of P caused the accumulation of PA in alfalfa silage, creating an acidic environment that effectively inhibited the growth of undesirable microorganisms and enhanced anaerobic fermentation. During silage fermentation, insufficient additives or unfavorable fermentation conditions may promote the growth of butyric acid-producing bacteria, leading to excessive BA, which can reduce the quality of forage silage and cause spoilage and deterioration (21). In our study, the BA content of the CK and LP groups was higher than that of the CL and P groups when the moisture content was 78%. This indicates that suitable additives can effectively reduce the production of BA without wilting. Studies have shown that the BA content decreases as the moisture content decreases (30). Flieg’s score is a commonly used indicator for evaluating the fermentation quality of silage (31). Our results showed that the Flieg’s score of the P group was higher than that of the CL and LP groups when the moisture content was 78%, similar to the CL and LP groups when the moisture content was 68%, but was lower than that of the CL and LP groups when the moisture content was 58%. A possible reason is that as the moisture content of alfalfa decreases, the number of beneficial microorganisms on its surface declines, while the growth of harmful microorganisms accelerates. This may weaken the effect of P while enhancing the effects of CL and LP. This is consistent with previous findings (14).

It has been reported that adding a chemical additive (a mixture of sodium nitrite, potassium sorbate, and sodium benzoate) and lactic acid bacteria (LP, *Bifidobacterium brevis,* and *Pediococcus pentosaceus*) can reduce the loss of DM and CP (32). In our study, we found that additives significantly reduced DM loss and CP degradation in alfalfa silage, indicating that appropriate additives can effectively reduce DM and CP losses during fermentation.

Many studies have reported that Firmicutes and Proteobacteria are the main bacterial phyla present in alfalfa silage (10, 33). In our study, Firmicutes and Proteobacteria were the predominant phyla, while the dominant genera were *Lactobacillus* and *Enterococcus*. This indicates that both high (without wilting) and low (with 58%) levels of moisture can promote the proliferation of harmful and miscellaneous bacteria, which is unfavorable for anaerobic fermentation of alfalfa. In our study, *Lactobacillus* was the dominant genus in the CL, LP, and P groups, except in the P group at the 58% moisture content, while *Enterococcus* abundance was lower in the additive groups than in the CK group. It has been reported that *Lactobacillus* is the dominant bacterial genus in well-fermented alfalfa silage (32). In contrast, *Enterococcus* was the dominant bacterial genus in the CK group at all moisture contents. In addition, *Lactobacillus* was negatively correlated with pH and Flieg’s score and positively correlated with LA. *Enterococcus* was negatively correlated with LA, Flieg’s score, and DM and positively correlated with pH. This is consistent with other studies reporting that *Enterococcus* is the dominant bacterial genus in poorly fermented alfalfa silage (13, 30).

The software PICRUSt2 was used to predict bacterial functions (34). Our analysis of KEGG pathways in alfalfa silage revealed that at Level 1, the metabolism pathway was the most active. Further analysis at Level 2 showed that carbohydrate and amino acid metabolism were the primary functional subcategories within this pathway. Specifically, the addition of LP significantly enhanced carbohydrate metabolism. This effect was dependent on moisture content: compared to the CK group, LP improved carbohydrate metabolism at 78% moisture; compared to the CL group, it showed improvement at 68% moisture; and compared to the P group, improvement was observed at 58% moisture. The results may indicate that LP improves the fermentation characteristics and nutritional quality of alfalfa by enhancing its carbohydrate metabolism. Furthermore, starch and sucrose metabolism was identified as the top pathway for carbohydrate metabolism at KEGG pathway level 3. In addition, the carbohydrate metabolism function of alfalfa silage treated with P appeared to be weakly active, suggesting that carbohydrate metabolism is more dominant in well-fermented silage.

## Conclusion

At moisture contents of 78 and 68%, the addition of CL, LP, and P significantly reduced the pH, NH_3_-N content, and *Enterococcus* abundance in alfalfa silage, while increasing the LA content, DM content, and *Lactobacillus* abundance. Moreover, at the 68% moisture content, all additives significantly increased the CP content. At the 58% moisture content, the addition of CL and LP significantly reduced the pH, NH_3_-N content, and the relative abundance of *Enterococcus* in alfalfa silage, while increasing the relative abundance of *Lactobacillus*. Functional prediction showed that additives improved metabolism at KEGG pathway level 1 in alfalfa silage, and this effect became more pronounced as the moisture content decreased. Carbohydrate metabolism was the most active pathway at KEGG pathway level 2, and the LP group exhibited the highest relative abundance. Moreover, starch and sucrose metabolism was the most active pathway at KEGG pathway level 3 in the LP-treated silage.

## Data Availability

The original contributions presented in the study are included in the article/Supplementary material, further inquiries can be directed to the corresponding author.

## Footnotes

1
https://cloud.majorbio.com
