# Supplementary material for: Moisture-driven shifts in the fermentation characteristics and microbial community of alfalfa silage treated with different additives
Source: Front Vet Sci. 2026 Feb 27;13:1748640. doi: 10.3389/fvets.2026.1748640 (PMC12982052; doi:10.3389/fvets.2026.1748640)
Supplement: Supplementary file 1 [file Table_1.DOCX]

**Table S1** The relative abundances of the top 10 phyla among four additive groups at different moisture contents.

| **Moistures** | **Phyla** | **CK-Mean(%)** | **CK-Sd(%)** | **CL-Mean(%)** | **CL-Sd(%)** | **LP-Mean(%)** | **LP-Sd(%)** | **P-Mean(%)** | **P-Sd(%)** | ***P*-value** |
| --- | --- | --- | --- | --- | --- | --- | --- | --- | --- | --- |
| **78%** | p__*Firmicutes* | 92.09 | 2.91 | 82.06 | 12.16 | 92.81 | 5.13 | 78.93 | 8.68 | 0.10 |
|  | p__*Proteobacteria* | 7.30 | 2.93 | 16.15 | 12.15 | 6.10 | 4.91 | 15.38 | 5.66 | 0.19 |
|  | p__*Cyanobacteria* | 0.28 | 0.07 | 0.56 | 0.03 | 0.46 | 0.26 | 4.13 | 3.06 | 0.05 |
|  | p__*Actinobacteriota* | 0.26 | 0.11 | 1.12 | 0.04 | 0.53 | 0.07 | 1.39 | 0.31 | 0.02 |
|  | p__*unclassified_k__norank_d__Bacteria* | 0.04 | 0.02 | 0.09 | 0.06 | 0.06 | 0.04 | 0.07 | 0.02 | 0.54 |
|  | p__*Bacteroidota* | 0.01 | 0.01 | 0.01 | 0.01 | 0.02 | 0.02 | 0.04 | 0.02 | 0.19 |
|  | p__*Bdellovibrionota* | 0.00 | 0.00 | 0.01 | 0.01 | 0.01 | 0.01 | 0.02 | 0.01 | 0.14 |
|  | p__*Verrucomicrobiota* | 0.00 | 0.00 | 0.00 | 0.00 | 0.01 | 0.00 | 0.01 | 0.00 | 0.07 |
|  | p__*Deinococcota* | 0.00 | 0.00 | 0.00 | 0.00 | 0.00 | 0.00 | 0.01 | 0.00 | 0.04 |
|  | p__*Planctomycetota* | 0.00 | 0.00 | 0.00 | 0.00 | 0.00 | 0.00 | 0.01 | 0.01 | 0.29 |
| **68%** | p__*Firmicutes* | 88.89 | 5.14 | 57.04 | 3.25 | 92.18 | 1.35 | 86.70 | 14.72 | 0.09 |
|  | p__*Proteobacteria* | 10.13 | 5.01 | 29.16 | 9.67 | 6.24 | 1.33 | 9.90 | 11.17 | 0.12 |
|  | p__*Cyanobacteria* | 0.56 | 0.42 | 11.17 | 12.08 | 0.83 | 0.38 | 2.34 | 2.49 | 0.07 |
|  | p__*Actinobacteriota* | 0.33 | 0.08 | 2.26 | 0.37 | 0.60 | 0.28 | 0.95 | 0.98 | 0.06 |
|  | p__*unclassified_k__norank_d__Bacteria* | 0.06 | 0.02 | 0.15 | 0.03 | 0.07 | 0.02 | 0.08 | 0.08 | 0.16 |
|  | p__*Bacteroidota* | 0.01 | 0.01 | 0.08 | 0.07 | 0.05 | 0.03 | 0.01 | 0.01 | 0.08 |
|  | p__*Bdellovibrionota* | 0.01 | 0.01 | 0.03 | 0.02 | 0.01 | 0.00 | 0.01 | 0.01 | 0.13 |
|  | p__*Verrucomicrobiota* | 0.00 | 0.00 | 0.03 | 0.03 | 0.01 | 0.01 | 0.00 | 0.00 | 0.22 |
|  | p__*Deinococcota* | 0.00 | 0.00 | 0.03 | 0.01 | 0.00 | 0.00 | 0.00 | 0.00 | 0.02 |
|  | p__*Planctomycetota* | 0.00 | 0.00 | 0.02 | 0.03 | 0.00 | 0.00 | 0.00 | 0.00 | 0.46 |
| **58%** | p__*Firmicutes* | 68.88 | 24.05 | 70.11 | 7.13 | 85.08 | 11.05 | 31.03 | 18.19 | 0.07 |
|  | p__*Proteobacteria* | 26.99 | 22.21 | 22.97 | 9.46 | 11.97 | 10.04 | 51.60 | 10.65 | 0.08 |
|  | p__*Cyanobacteria* | 3.26 | 1.73 | 4.83 | 2.87 | 1.23 | 0.70 | 14.27 | 6.72 | 0.03 |
|  | p__*Actinobacteriota* | 0.71 | 0.18 | 1.78 | 0.57 | 0.69 | 0.16 | 2.79 | 1.22 | 0.03 |
|  | p__*Bacteroidota* | 0.06 | 0.05 | 0.06 | 0.03 | 0.88 | 1.47 | 0.15 | 0.07 | 0.33 |
|  | p__*unclassified_k__norank_d__Bacteria* | 0.07 | 0.01 | 0.16 | 0.05 | 0.12 | 0.06 | 0.11 | 0.07 | 0.18 |
|  | p__*Deinococcota* | 0.00 | 0.00 | 0.03 | 0.02 | 0.02 | 0.01 | 0.01 | 0.00 | 0.06 |
|  | p__*Bdellovibrionota* | 0.01 | 0.00 | 0.03 | 0.02 | 0.01 | 0.00 | 0.02 | 0.01 | 0.22 |
|  | p__*Verrucomicrobiota* | 0.00 | 0.00 | 0.02 | 0.01 | 0.00 | 0.00 | 0.01 | 0.01 | 0.02 |
|  | p__*Planctomycetota* | 0.01 | 0.01 | 0.01 | 0.00 | 0.00 | 0.00 | 0.00 | 0.00 | 0.31 |

**Table S2** The relative abundances of the top 10 genera among four additive groups at different moisture contents.

| **Moistures** | **Genera** | **CK-Mean(%)** | **CK-Sd(%)** | **CL-Mean(%)** | **CL-Sd(%)** | **LP-Mean(%)** | **LP-Sd(%)** | **P-Mean(%)** | **P-Sd(%)** | ***P*-value** |
| --- | --- | --- | --- | --- | --- | --- | --- | --- | --- | --- |
| 78% | g__*Lactobacillus* | 8.30 | 3.23 | 78.08 | 12.37 | 88.63 | 4.43 | 71.76 | 7.52 | 0.03 |
|  | g__*Enterococcus* | 65.75 | 13.75 | 2.13 | 1.12 | 0.46 | 0.11 | 3.14 | 2.26 | 0.02 |
|  | g__*Allorhizobium-Neorhizobium-Pararhizobium-Rhizobium* | 0.73 | 0.14 | 7.02 | 9.08 | 1.43 | 1.16 | 2.91 | 1.81 | 0.13 |
|  | g__*Methylobacterium-Methylorubrum* | 1.44 | 0.41 | 2.22 | 0.58 | 1.12 | 0.76 | 3.33 | 0.72 | 0.05 |
|  | g__*Aureimonas* | 0.50 | 0.16 | 2.70 | 1.45 | 1.01 | 0.75 | 3.11 | 1.50 | 0.04 |
|  | g__*unclassified_o__Lactobacillales* | 4.70 | 0.69 | 0.70 | 0.26 | 0.25 | 0.03 | 1.10 | 0.73 | 0.02 |
|  | g__*Leuconostoc* | 5.06 | 3.62 | 0.02 | 0.02 | 0.50 | 0.84 | 0.26 | 0.13 | 0.07 |
|  | g__*norank_f__norank_o__Chloroplast* | 0.28 | 0.07 | 0.56 | 0.03 | 0.46 | 0.26 | 4.13 | 3.06 | 0.05 |
|  | g__*Enterobacter* | 3.79 | 2.43 | 0.89 | 0.86 | 0.32 | 0.30 | 0.33 | 0.25 | 0.10 |
|  | g__*Pediococcus* | 0.63 | 0.40 | 0.90 | 0.57 | 2.43 | 0.69 | 1.24 | 1.52 | 0.19 |
| 68% | g__*Lactobacillus* | 25.53 | 7.38 | 55.68 | 3.54 | 91.86 | 1.36 | 73.03 | 14.82 | 0.02 |
|  | g__*Enterococcus* | 54.17 | 8.00 | 0.64 | 0.22 | 0.16 | 0.06 | 4.83 | 1.05 | 0.02 |
|  | g__*norank_f__norank_o__Chloroplast* | 0.56 | 0.42 | 11.17 | 12.08 | 0.83 | 0.37 | 2.33 | 2.49 | 0.07 |
|  | g__*Methylobacterium-Methylorubrum* | 3.60 | 2.24 | 6.19 | 2.01 | 0.95 | 0.06 | 2.22 | 2.43 | 0.09 |
|  | g__*Aureimonas* | 1.05 | 0.65 | 7.55 | 3.05 | 1.52 | 0.18 | 1.88 | 1.62 | 0.08 |
|  | g__*Weissella* | 3.22 | 1.91 | 0.22 | 0.07 | 0.05 | 0.03 | 5.99 | 7.50 | 0.02 |
|  | g__*Allorhizobium-Neorhizobium-Pararhizobium-Rhizobium* | 1.62 | 1.81 | 4.51 | 2.96 | 1.17 | 0.56 | 1.61 | 1.68 | 0.28 |
|  | g__*Sphingomonas* | 0.45 | 0.15 | 4.64 | 3.08 | 0.77 | 0.21 | 2.38 | 3.40 | 0.14 |
|  | g__*unclassified_o__Lactobacillales* | 4.65 | 0.54 | 0.22 | 0.04 | 0.06 | 0.03 | 2.51 | 0.12 | 0.02 |
|  | g__*Enterobacter* | 2.55 | 3.53 | 0.70 | 0.39 | 0.12 | 0.02 | 0.37 | 0.58 | 0.17 |
| 58% | g__*Lactobacillus* | 22.61 | 18.09 | 69.64 | 6.83 | 84.82 | 11.20 | 28.00 | 17.85 | 0.03 |
|  | g__*Enterococcus* | 37.47 | 10.26 | 0.20 | 0.21 | 0.11 | 0.07 | 2.47 | 0.27 | 0.02 |
|  | g__*Methylobacterium-Methylorubrum* | 7.38 | 1.45 | 3.97 | 0.70 | 1.41 | 0.35 | 19.68 | 7.53 | 0.02 |
|  | g__*norank_f__norank_o__Chloroplast* | 3.26 | 1.73 | 4.82 | 2.86 | 1.22 | 0.70 | 14.27 | 6.73 | 0.03 |
|  | g__*Sphingomonas* | 1.17 | 0.40 | 4.79 | 2.68 | 3.81 | 4.21 | 11.19 | 2.11 | 0.04 |
|  | g__*Aureimonas* | 2.26 | 0.76 | 5.46 | 3.19 | 2.20 | 0.98 | 7.17 | 0.92 | 0.04 |
|  | g__*Enterobacter* | 12.59 | 21.35 | 1.15 | 1.57 | 0.25 | 0.34 | 1.01 | 0.84 | 0.46 |
|  | g__*Allorhizobium-Neorhizobium-Pararhizobium-Rhizobium* | 1.61 | 0.14 | 3.96 | 2.70 | 1.12 | 0.91 | 7.05 | 0.85 | 0.03 |
|  | g__*unclassified_o__Lactobacillales* | 4.46 | 2.41 | 0.06 | 0.05 | 0.08 | 0.07 | 0.38 | 0.05 | 0.02 |
|  | g__*Curtobacterium* | 0.38 | 0.07 | 0.91 | 0.31 | 0.38 | 0.15 | 1.76 | 1.25 | 0.03 |


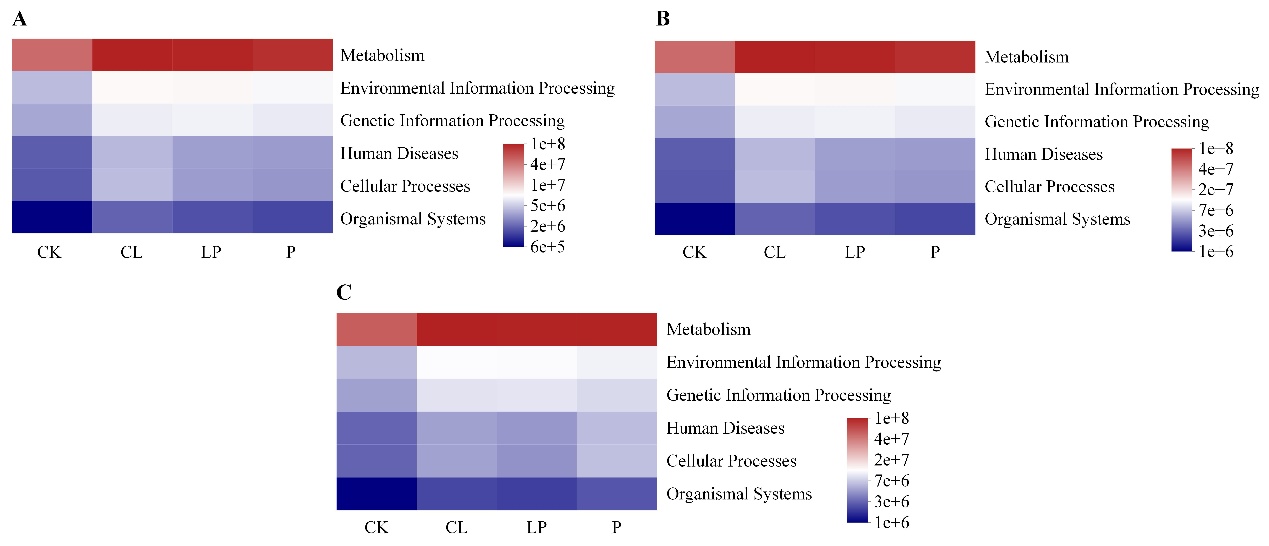


**FIGURE S1 ⎢** The relative abundance of function KEGG pathway level 1 of alfalfa silage bacteria at the moisture content of 78% (**A**), 68% (**B**) and 58% (**C**).

CK, group with an equal amount of distilled water as control check; CL, group inoculated with commercial *Lactobacillus plantarum* group; LP, group inoculated with *L. plantarum*; P, group added with propionic-acid.


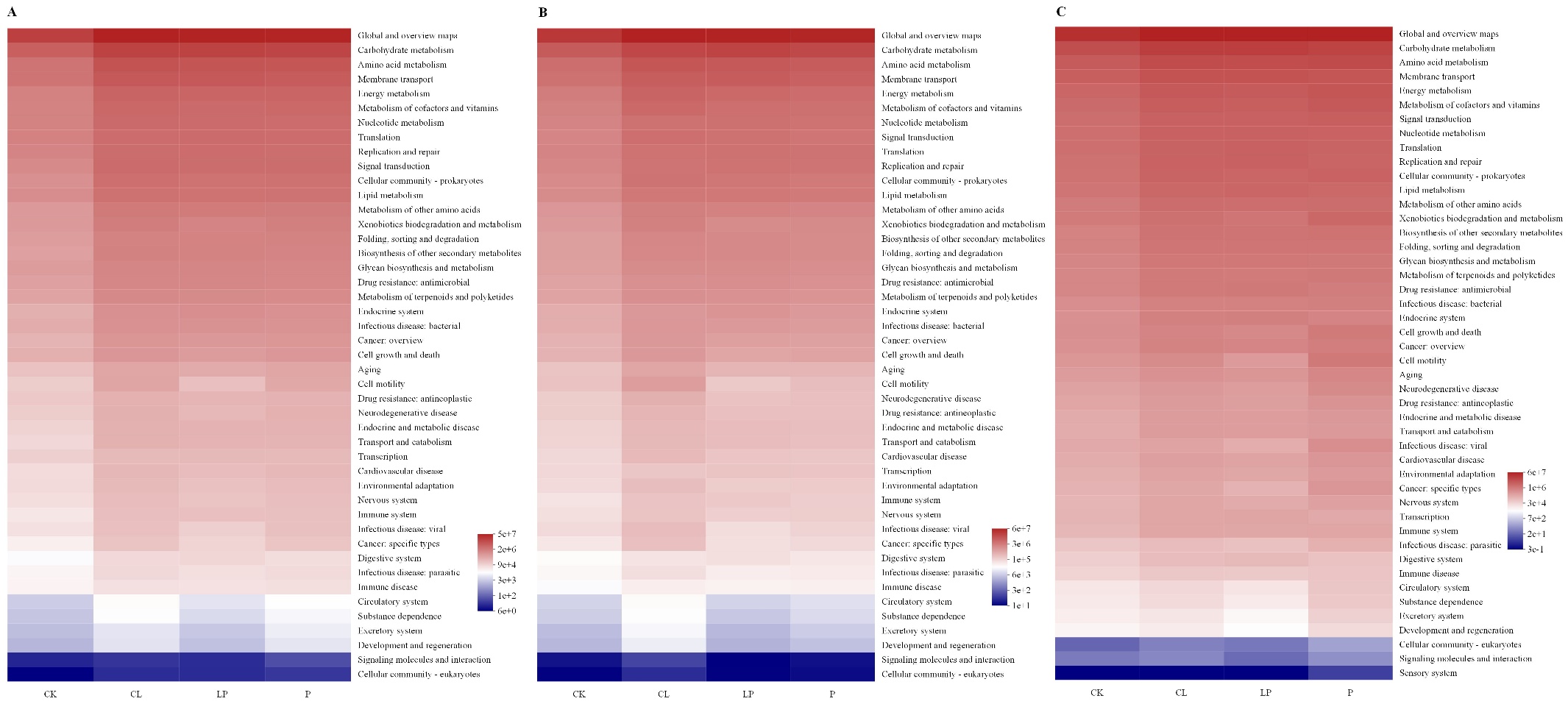


**Fig.** **S2** The relative abundance of function KEGG pathway level 2 of alfalfa silage bacteria at the moisture content of 78% (**A**), 68% (**B**) and 58% (**C**). CK, group with an equal amount of distilled water as control check; CL, group inoculated with commercial *Lactobacillus plantarum* group; LP, group inoculated with *L. plantarum*; P, group added with propionic-acid.


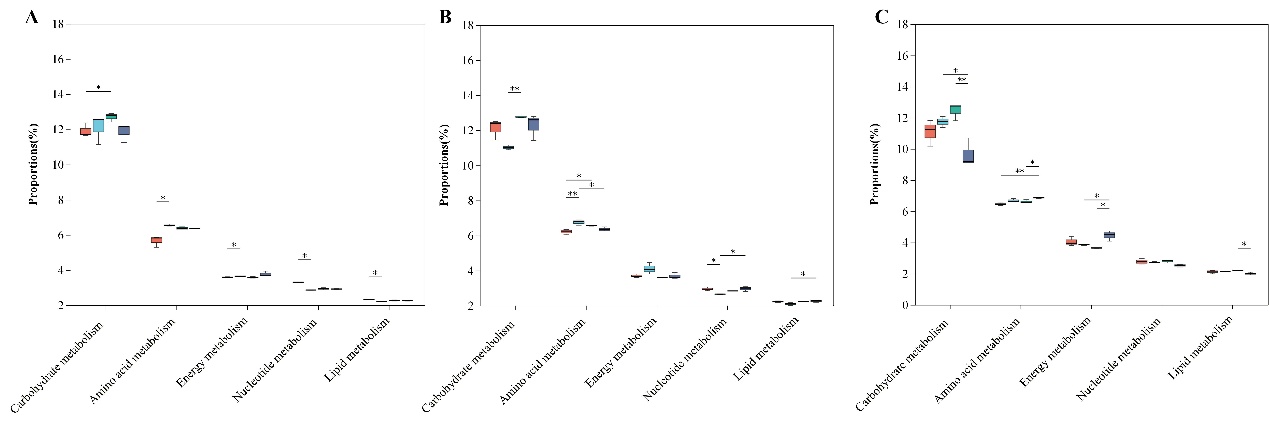


**FIGURE S3 ⎢** Box plot of functional predictions differences of alfalfa silage treated with different additives at different moisture content.


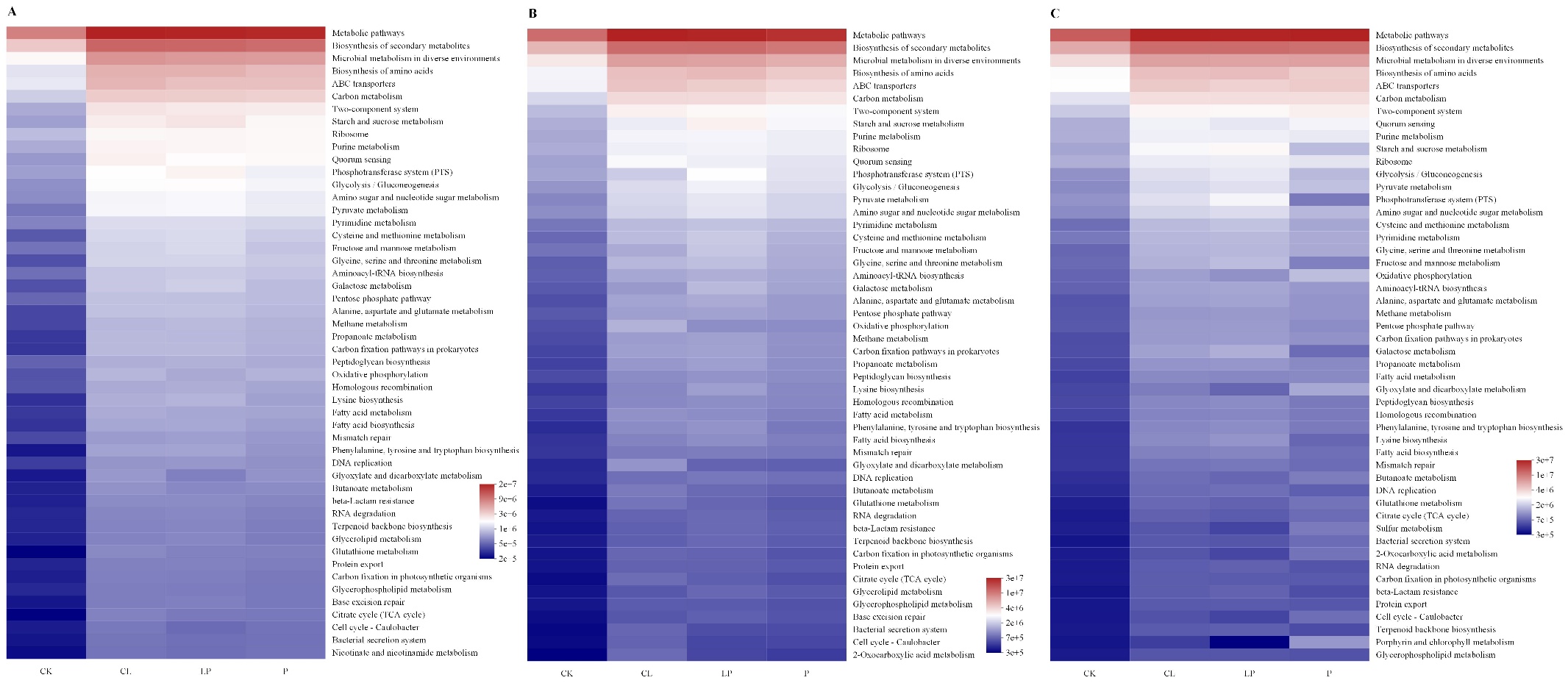


**FIGURE S4** The relative abundance of function KEGG pathway level 3 of alfalfa silage bacteria at the moisture content of 78% (**A**), 68% (**B**) and 58% (**C**). CK, group with an equal amount of distilled water as control check; CL, group inoculated with commercial *Lactobacillus plantarum* group; LP, group inoculated with *L. plantarum*; P, group added with propionic-acid.


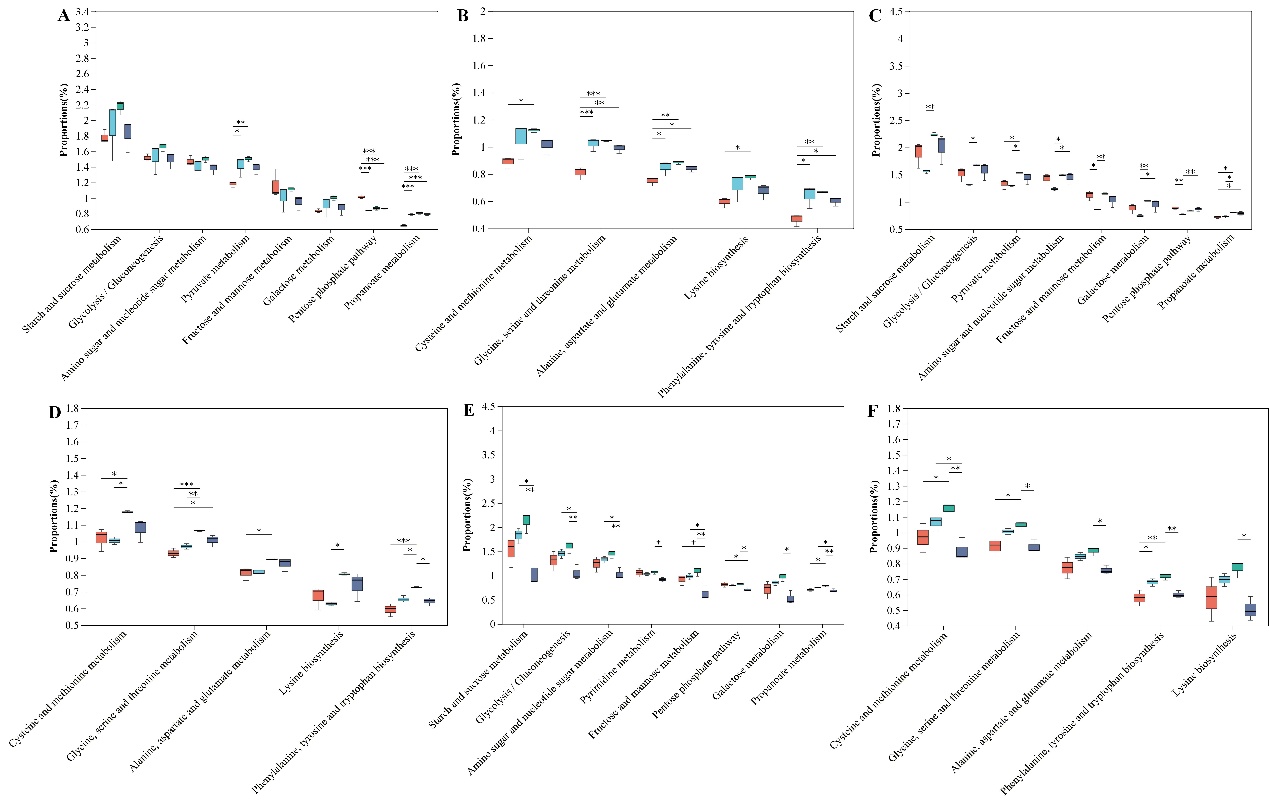


**FIGURE S5 ⎢** Box plot of functional predictions differences of alfalfa silage treated with different additives at different moisture content.

Relative abundance of function KEGG pathway level 3, carbohydrate metabolism (**A**), amino acid metabolism (**B**) at the moisture content of 78%, carbohydrate metabolism (**C**), amino acid metabolism (**D**), at the moisture content of 68%, and carbohydrate metabolism (**E**), amino acid metabolism (**F**), at the moisture content of 58%. CK, group with an equal amount of distilled water as control check; CL, group inoculated with commercial *Lactobacillus plantarum* group; LP, group inoculated with *L. plantarum*; P, group added with propionic-acid.
